# Fear of Bioterrorism and Implications for Public Health Preparedness

**DOI:** 10.3201/eid0904.020593

**Published:** 2003-04

**Authors:** Mark S. Dworkin, Xinfang Ma, Roman G. Golash

**Affiliations:** * Illinois Department of Public Health, Chicago, Illinois, USA

**Keywords:** anthrax, bioterrorism, Bacillus anthracis, dispatch

## Abstract

After the human anthrax cases and exposures in 2001, the Illinois Department of Public Health received an increasing number of environmental and human samples (1,496 environmental submissions, all negative for *Bacillus anthracis*). These data demonstrate increased volume of submissions to a public health laboratory resulting from fear of bioterrorism.

After the terrorist attacks of September 11, 2001, and the discovery of human anthrax cases and exposures several weeks later on the East Coast, Illinois Department of Public Health’s (IDPH) Division of Laboratories received environmental and human samples for analysis as part of suspected bioterrorism investigations. The large number of samples provided an opportunity to learn about the possible presence of *Bacillus anthracis* in the environment, to gain insight into the frequency of true versus perceived bioterrorist events, and to observe and respond to the impact of widely publicized terrorism alerts.

## The Study

Submissions of powder and nonpowder environmental samples and human blood or tissue specimens submitted from October 8 through December 31, 2001, were reviewed. Before samples were accepted by IDPH Division of Laboratories, incidents involving environmental samples had to be reviewed by Federal Bureau of Investigations (FBI) agents who determined if a potential bioterrorism threat was credible. The IDPH laboratories in Chicago and Springfield processed all samples submitted through law enforcement authorities in this manner. The Chicago laboratory received samples primarily from northern Illinois (north of Interstate 80), while the Springfield laboratory received samples from central and southern Illinois. The Chicago laboratory followed all required guidelines for a biosafety level 3 laboratory (*1*).

The laboratory methods for identifying *Bacillus* species in environmental samples have been reported elsewhere (*2*). Laboratory methods included Gram stain and culture of suspicious colonies grown on blood agar plates, and beta-lactamase, motility, and gamma-phage lysis testing. A malachite green stain for spores was performed on all powder specimens during the initial 2 weeks at the Chicago laboratory and on selected specimens thereafter; an M’Fadyean stain was used at the Springfield laboratory. Polymerase chain reaction (PCR) was performed on those samples requiring the most rapid turnaround time (e.g., specimens submitted by a U.S. Postal Service facility that had been closed pending results). Human samples arrived from hospital laboratories in the form of a tryptic soy agar slant and were plated to blood agar plates upon arrival. Gamma-phage lysis, PCR, or both were performed as needed. The Chicago and Springfield laboratories’ processed their first samples on October 8 and October 9, respectively.

Because no data were available regarding what to expect from processing bioterrorism threat-related samples from the environment, *Bacillus* organisms from most environmental and human samples were speciated, even if negative for *B. anthracis*.

### Environmental Specimens

A total of 1,496 environmental specimens were processed: 1,193 (79.7%) in Chicago and 303 (20.3%) in Springfield. An additional 40 human specimens were processed, 28 (70%) in Chicago and 12 (30%) in Springfield. Chicago sample submissions rose steadily after the first week of October and peaked during the week of October 29 through November 4, with the largest number of submissions processed on November 7 (range 0–64 submissions per day) (Figure). An additional 17 submissions for which the date of submission was not clearly documented, and may have preceded October 8, also were processed. Powdery substances constituted 42.0% of submissions to the Chicago laboratory versus 33.7% of submissions to the Springfield laboratory. Nonpowdery substances (e.g., environmental swab samples, letters, envelopes, packages, and other materials) constituted 58.0% of submissions to the Chicago laboratory versus 66.3% of submissions to the Springfield laboratory. Eight additional environmental samples that did not go through the FBI were received by the Chicago laboratory from hospitals.

**Figure:**
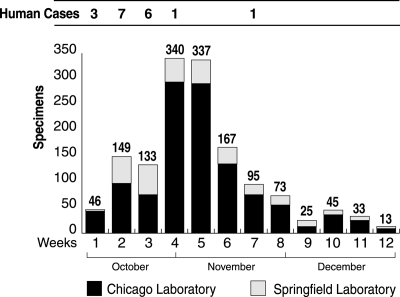
Number of environmental specimens submitted to the Illinois Department of Public Health Division of Laboratories for *Bacillus anthracis* testing each week from October 8 through December 30, 2001 and number of human cases occurring on the East Coast and reported each week in the news media.

Among both the powders and nonpowders processed at the Chicago laboratory, the most frequently isolated organisms were *Bacillus cereus* (19.2% and 8%, respectively) and nonhemolytic staphylococci (23.9% and 22.8%, respectively) (Table 1). Among the eight hospital samples not submitted through the FBI, *B. megaterium* (four), *B. thuringiensis* (one), and nonanthracis *Bacillus* species (three) were identified. Twenty specimens (8 in Chicago and 12 in Springfield) were processed by using PCR. All of these results were negative for *B. anthracis*.

**Table 1:** Results of 1,364 environmental specimens submitted to the Illinois Department of Public Health Division of Laboratories (Chicago laboratory) for *Bacillus anthracis* testing, October–December 2001

Organism	Powder (%)	Nonpowder (%)
Total	573 (42.0)	791 (58.0)
*B. cereus*	110 (19.2)	63 (8.0)
*B. subtilis*	29 (5.1)	32 (4.0)
Other *Bacillus* species (hemolytic, positive motility)	21 (3.7)	40
*B. mycoides*	16 (2.8)	6
*B. circulans*	10 (1.7)	4
*B. pumilus*	2 (0.3)	5
*B. brevis*	1 (0.2)	1
*B. laterosporus*	0	1
*B. megaterium*	0	1
*B. polymyxa*	0	1
*B. anthracis*	0	0
*Staphylococcus* species (nonhemolytic)	137 (23.9)	180
*Staphylococcus aureus*	3 (0.5)	5
Other mixed gram-positive organisms	48 (8.3)	68
Gram-negative bacilli	80 (14.0)	72
*Enterobacter agglomerans*	1 (0.2)	0
*Moraxella catarrhalis*	1 (0.2)	0
Mixed other gram-positive organisms and mold	8 (1.4)	11
Mold	19 (3.3)	32
No growth	87 (15.2)	269

A review of the source and circumstances related to 57 nonhuman samples submitted to the Chicago laboratory on November 7, 2001 (the date with the highest number of submissions), indicated that most of the items were mail items (e.g., 18 letters or envelopes, 12 unspecified mail items, and 7 packages), but powders (7 submissions) and unspecified “suspicious substance” (13 items) also were identified. Such items came from at least six counties and involved 13 local police departments, the Illinois State Police, two fire departments, and the FBI. In addition to submissions from private citizens, submissions came from two universities, a post office, a private business, and a consulate. A high level of anxiety among the public was likely responsible for the otherwise unsuspicious items submitted, including dairy creamer, powder from donuts, a backpack, a telephone, a frozen dinner, a computer keyboard, and a letter from a married man’s lover that was intercepted by his wife and submitted unopened as a suspicious mail item.

### Human Specimens

Twenty-eight human specimens were submitted to the Chicago state laboratory for evaluation after preliminary testing at an initial laboratory (usually a hospital) could not rule out *B. anthracis* (Table 2). These included 15 blood cultures. For 12 specimens, the species were not identified, although test results demonstrated they were not any of 16 *Bacillus* species. An additional 12 specimens submitted to the Springfield laboratory were negative for *B. anthracis*.

**Table 2:** Results of 28 human specimens submitted to the Illinois Department of Public Health Division of Laboratories (Chicago laboratory) for *Bacillus anthracis* testing, October–December 2001^a^

Organism	No. (%)	Source (no.)
*B. cereus*	5 (17.9)	Blood (3), nasal (2)
*B. megaterium*	4 (14.3)	Blood (2), leg wound (1), abdominal fluid (1)
*B. subtilis*	2 (7.1)	Blood (2)
*B. brevis*	1 (3.6)	Wound (1)
*B. coagulans*	1 (3.6)	Blood (1)
*B. firmus*	1 (3.6)	Blood (1)
*B. pumulus*	1 (3.6)	Blood (1)
*Paenibacillus macerans*	1 (3.6)	Unspecified
*Bacillus* species, other (not speciated)	12 (42.9)	Blood (8), nasal (1), body fluid (1), cerebrospinal fluid (1), unspecified (1)
*B. anthracis*	—	—

^a^After preliminary testing at an Illinois laboratory could not rule out *B. anthracis*.

We found no samples positive for *B. anthracis* among the nearly 1,500 submissions to IDPH for testing. Other state health departments also received large numbers of submissions: Michigan and Oklahoma received at least 228 and 762 submissions, respectively (*3*,*4*). These results demonstrate the potential stress to a public health laboratory that may result from bioterrorism-related anxiety and hyperalertness to the environment. This high state of alert by the public is important for early recognition of a real bioterrorism event through enhanced reporting.

Our data demonstrate that the number of submissions was temporally associated with the media attention to anthrax-related events in Florida, New York City, Washington, D.C., and other affected areas. Concern was reinforced with messages from government, warning of the need for the public to be on heightened alert for terrorism. On October 29, 2001, the U.S. attorney general and the FBI director announced that U.S. citizens and law enforcement agencies should be on “highest alert” based on “credible” information, and police and citizens should be “extremely vigilant”. Forty-five percent of the nearly 1,500 environmental specimens submitted to the state laboratory for testing during the 12-week period arrived during the 2 weeks after that announcement, which created an unprecedented workload in bioterrorism evaluation.

During the surge in laboratory demand, adequate numbers of trained personnel to process the large volume of submissions were needed. Additional staff were trained, and work hours were expanded. Despite these efforts, a backlog occurred, causing specimens to be grouped in order of priority for rapid versus delayed processing. Delay in processing added to the anxiety experienced by many who submitted samples. Laboratory staff were distracted by frequent requests for updated information. Because of evolving information, emerging guidance from the Centers for Disease Control and Prevention, and the difficulty of managing staff who were being pulled away from other laboratory jobs to assist with bioterrorism samples, meetings were held frequently (often more than once per day). However, such meetings also competed with laboratory time, which was needed to process the increasing load of submissions.

Among the more positive aspects of the high volume of submissions was enhanced cooperation with law enforcement officials, especially the FBI. Such officials maintained a temporary office in the Chicago laboratory facility, where submissions could be received at all hours, prioritized, and then delivered individually or batched for submission to the laboratory (which did not have a night shift of workers) during working hours.

## Conclusions

Because health department laboratories have previously had only limited and sporadic experience with the test methods involved in urgent microbiologic evaluation for anthrax, the experience with these submissions provided an opportunity to polish critical laboratory skills, to utilize infrequently used or not previously available equipment (e.g., real time PCR), to speciate nonanthracis *Bacillus* isolates to determine what species may be expected during such an event, and to anticipate what personnel and changes in protocols might be needed during future bioterrorist events. This experience also highlighted the importance of regular training and updating of laboratory staff on procedures relevant to bioterrorism agent evaluation.

We recommend continued close communication and collaboration between public health and law enforcement officials, which include developing flexible criteria on specimen submission guidelines for public laboratories. Development of standardized forms for information collection related to suspicious substance submissions may be useful in evaluating the epidemiology of any future bioterrorism events. We also recommend regular training of laboratory staff on procedures relevant to bioterrorism agent evaluation, including cross-training of selected staff not usually involved in the handling of suspected bioterrorism agents as part of a surge capacity plan. Such a plan should also include a mechanism for handling submissions after regular work hours because of the high profile and expectations of turnaround time that submissions for bioterrorism agent evaluation usually have. Finally, enhancing communication between the many agencies involved remains both a challenge and an opportunity for future efforts to combat these kinds of events.
